# Copy number variations and expression of MPDZ are prognostic biomarkers for clear cell renal cell carcinoma

**DOI:** 10.18632/oncotarget.20220

**Published:** 2017-08-12

**Authors:** Yong-Sheng Huang, Wen-Bin Liu, Fei Han, Jun-Tang Yang, Xiang-Lin Hao, Hong-Qiang Chen, Xiao Jiang, Li Yin, Lin Ao, Zhi-Hong Cui, Jia Cao, Jin-Yi Liu

**Affiliations:** ^1^ Institute of Toxicology, College of Preventive Medicine, Third Military Medical University, Chongqing 400038, PR China

**Keywords:** MPDZ, CNV, ccRCC, prognosis

## Abstract

The vital copy number variation (CNV) plays a crucial role in clear cell renal cell carcinoma (ccRCC). MPDZ inhibit cell polarity associate with osmotic pressure response and cancer-related biological processes. In order to clarify the role of the CNV of *MPDZ* in the progression of ccRCC, we analyzed the CNV and expression of *MPDZ* and prognosis in ccRCC patients from The Cancer Genome Atlas data portal. Notably, we found that the deletion of *MPDZ* was the common CNV, which was present in 28.65% of ccRCC patients. With the development of tumors, the percentage of *MPDZ* deletion increased significantly (19.38% in stage I; 20.00% in stage II; 40.94% in stage III; and 45.00% in stage IV). The deletion of *MPDZ* significantly increased ccRCC risk (P=0.0025). Low *MPDZ* expression associated with its deletion was significantly associated with adverse outcomes in ccRCC patients (P=0.0342). Furthermore, immunohistochemical analysis by tissue microarray showed that MPDZ was expressed at lower levels in tumor tissues compared with adjacent tissues (P<0.01). Kaplan–Meier survival curves showed that ccRCC patients with low MPDZ expression had significantly shorter survival than those with high MPDZ expression (P=0.002). These results indicated that low MPDZ expression associated with CNV is a potential biomarker for the prognosis of ccRCC patients.

## INTRODUCTION

Clear cell renal cell carcinoma (ccRCC), the most common and aggressive histologic subtype of RCC [1], is a malignant kidney neoplasm that may be fatal. It is estimated that approximately 55% of RCC would be fatal within 5 years after traditional treatment [2-4], because patients are often clinically diagnosed in the advanced phase of the disease. Recently, several novelty methods which primarily rely on clinical features [5-8] and several molecular biological markers [9-12] have blossomed and been externally validated to predict the progression of cancer with metastatic disease after surgical treatment. These morphologic risk criteria are fairly simplistic to facilitate the early diagnosis of ccRCC. Some genes CNVs, especially gene deletions, have been demonstrated to affect the overall clinical outlook of ccRCC and have been used for tumor diagnosis. However, it is still limited for the early prediction of ccRCC diagnosis. Therefore, it is urgent to identify and verify novel tumor molecular markers for early diagnosis, prognosis and therapy of ccRCC.

The human *multi-PDZ domain protein* (*MPDZ/MUPP1*) gene is located on chromosome 9p22-p24 [13]. Its full-length cDNA (EMBL accession number NM_003829) has an overall length of 7722 bp and encodes a protein of 2070 amino acids containing thirteen PDZ domains (Supplementary Figure 1A). As a tight junction-associated protein, MPDZ participates in the formation of connections between epithelial cells and endothelial cells, inhibits cell polarity and is closely related to the cell’s osmotic pressure response [14-16]. Although the function of *MPDZ* in tumorigenesis has rarely been reported, previous research found that MPDZ was involved in the carcinogenic effect of viruses as an interaction partner for the coxsackievirus and adenovirus receptor cytoplasmic domain [17]. The expression of *MPDZ* in breast cancer tissue was substantially lower than that in the normal mucosa and is correlated with cancer progression and aggression [18]. Clinical association analyses indicated that *MPDZ* deletion is related to poor survival in nasopharyngeal carcinoma [19]. However, the clinical relevance of *MPDZ* genetic alterations in ccRCC has not been addressed.

Osmotic pressure is an important factor in ccRCC progression. *MPDZ* is especially significant in the renal osmoadaptive response [15, 20]. It is reasonable to presume that *MPDZ* may play an important role in ccRCC. In the present study, we found that the deletion of *MPDZ* was frequently detected in ccRCC patients and the deletion of *MPDZ* was negatively correlated with its transcriptional expression. We also found that both the deletion of *MPDZ* and the expression of *MPDZ* were significantly associated with poor outcomes in patients with ccRCC. The molecular link between the deletion of *MPDZ* and cancer-specific outcomes suggests that *MPDZ* is a potential tumor suppressor gene in ccRCC. It provides a novel tumor molecular marker for diagnosis, prognostic and therapeutic purposes for patients with ccRCC.

## RESULTS

### The CNV of *MPDZ* frequently co-occurs in ccRCC patients from the TCGA cohort

**Figure 1:**
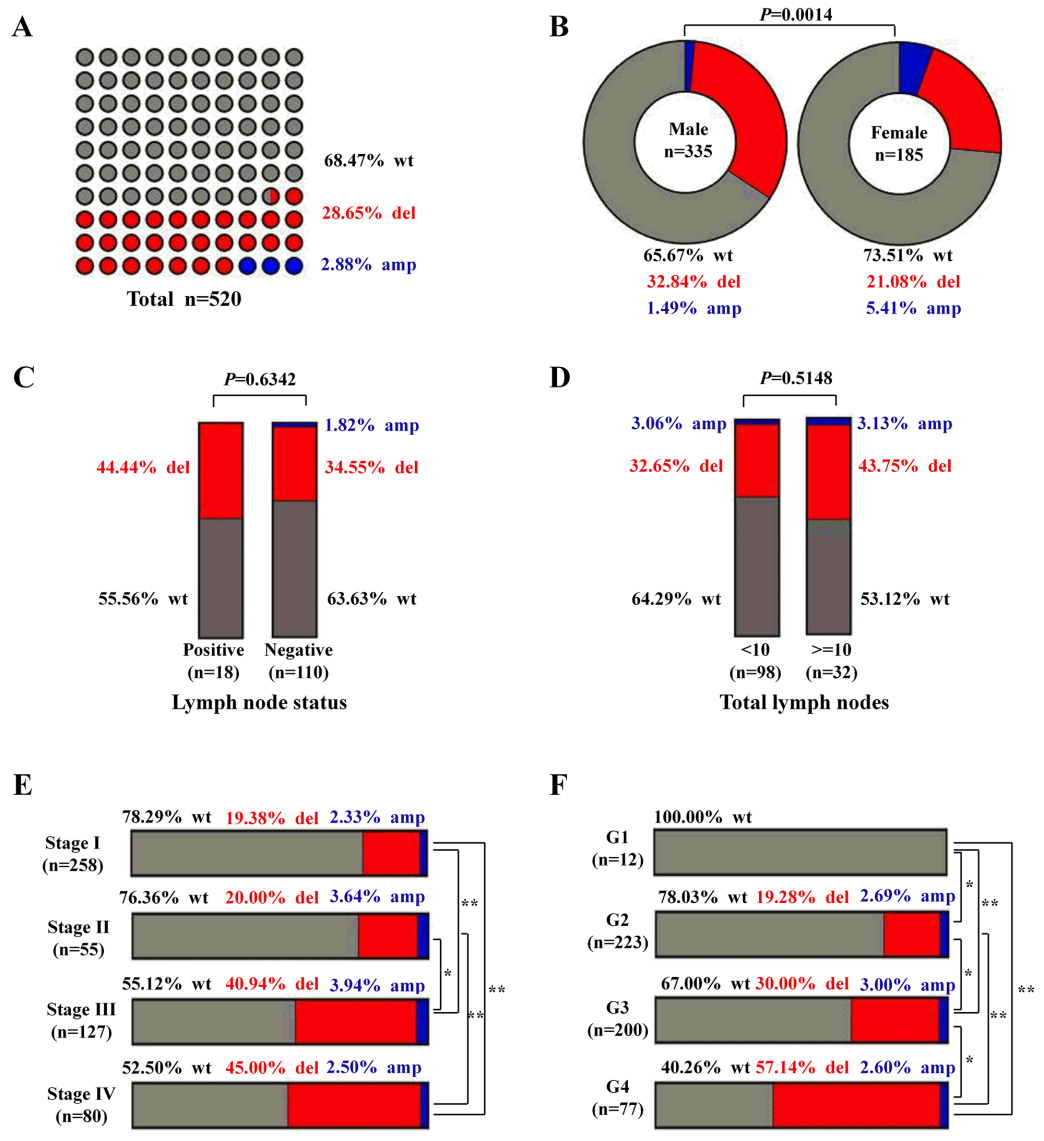
The CNV of MPDZ was frequently detected in ccRCC patients from the TCGA Cohort **(A)** The percentage of wild-type, deletions and amplifications in ccRCC, wt, wild-type; del, deletions; amp, amplifications. **(B)** Different percentage wild-type, deletions and amplifications in genders. **(C)** Different percentage of wild-type, deletions and amplifications in different lymph node status. **(D)** Different percentage of wild-type, deletions and amplifications in different total lymph nodes group. **(E)** Different percentage of wild-type, deletions and amplifications in different clinical stages. **(F)** Different percentage of wild-type, deletions and amplifications in different Fuhrman nuclear grades. The frequencies of deletions between different groups were used Chi-square test, Multiple comparisons that a 2 × 2 contingency table of expected and observed deletion frequencies were used to analyze more than two groups, with a P-value cut-off set at 0.05 with stringent false discovery rate control (Bonferroni’s method). * P<0.05, **P<0.01.

To evaluate the impact of the CNV of *MPDZ* on clinical outcomes, we assembled datasets from TCGA. The CNV of *MPDZ* was frequently detected in ccRCC patients as follows: 28.65% of deletions and 2.88% of amplifications (Figure 1A). The same distribution of CNV was performed for total lymph nodes and lymph node status (Figure 1C–1D), but not for gender (P=0.0014; Figure 1B). However, with an increase of clinical stage, the percentage of CNV also significantly increased as follows: 19.38% of deletions and 2.33% of amplifications in stage I; 20.00% of deletions and 3.64% of amplifications in stage II; 40.94% of deletions and 3.94% of amplifications in stage III; and 45.00% of deletions and 2.50% of amplifications in stage IV (Figure 1E). The similar distribution of *MPDZ* CNV was seen in different Fuhrman nuclear grades (Figure 1F).

### The deletion of *MPDZ* is associated with pathologic features in ccRCC patients from the TCGA cohort

Because gene deletion may lead to adverse outcomes in ccRCC, we further analyzed the relationship between the deletion of *MPDZ* and pathologic features. The relationship between the deletion of *MPDZ* and pathologic features that correlate with adverse outcomes are listed in Table 1. We found that the deletion of *MPDZ* was significantly correlated with gender (P=0.0096), Fuhrman nuclear grade (P<0.0001), AJCC stage (P<0.0001), pathological T stage (P<0.0001) and pathological M stage (P=0.0015), but not with age, pathological N stage, total lymph nodes and lymph node status. A subgroup analysis by pathological T stage revealed that the deletion of *MPDZ* in T1 was significantly different from T2, T3 and T4 (P<0.05), but not in any other two groups.

**Table 1:** Clinical factor and MPDZ copy number in ccRCC patients

Clinical factor	Numbers	MPDZ copy number status		χ2	*P* value
		Wt	Deletion		
**Total**	505	356	149		
***Age (year)***				3.52	0.0608
< 60	229	171	58		
≥ 60	276	185	91		
***Gender***				6.71	0.0096
male	330	220	110		
female	175	136	39		
***Total lymph nodes***				1.33	0.2491
< 10	95	63	32		
≥ 10	31	17	14		
Unknown	379	276	103		
***Lymph node status***				0.23	0.6324
Negative	108	70	38		
Positive	17	10	7		
Unknown	380	276	104		
***Nuclear grade***				23.51	< 0.0001
1+2	229	186	43		
3+4	269	165	104		
other	7	5	2		
***AJCC stage***				33.45	<0.0001
I+II	305	244	61		
III+IV	200	112	88		
***Pathological type***					
T (primary tumor)				37.92	<0.0001
T1	255	210	45		
T2	66	44	22		
T3	174	97	77		
T4	10	5	5		
N (regional lymph nodes)				1.95	0.3782
N0	225	164	61		
N1	17	10	7		
NX	263	182	81		
M (distant metastases)				13.05	0.0015
M0	409	292	117		
M1	73	42	31		
MX	23	22	1		

### The deletion of *MPDZ* is significantly associated with poor outcomes in ccRCC patients from the TCGA cohort

**Figure 2:**
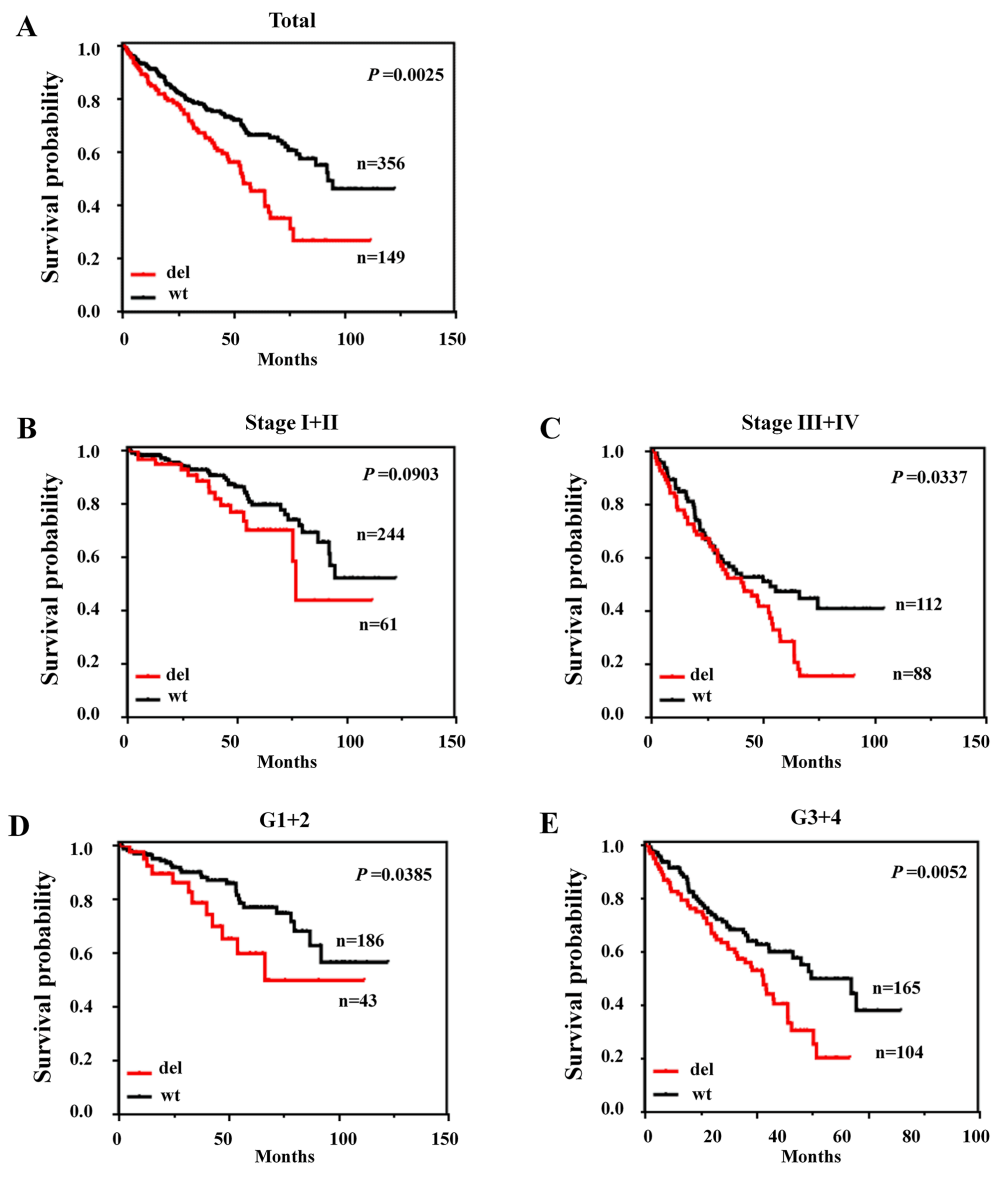
The deletion of MPDZ was associated with poor outcomes in ccRCC from the TCGA Cohort **(A)** Kaplan-Meier survival curves show that patients with *MPDZ* deletions had poorer survival than those with wild-type *MPDZ*. **(B)** Kaplan-Meier curves of deletions in patients with AJCC stage I and II. **(C)** Kaplan-Meier curves of deletions in patients with AJCC stage III and IV. **(D)** Kaplan-Meier curves of deletions in patients with Fuhrman nuclear grade 1 and 2. **(E)** Kaplan-Meier curves of deletions in patients with Fuhrman nuclear grade 3 and 4.

Due to the deletion of *MPDZ* was significantly correlated with Fuhrman nuclear grade and AJCC stage, the competing risk model was applied to survival analysis. The deletion of *MPDZ* is significantly associated with poor outcomes in ccRCC patients (P=0.0025; Figure 2A). A subgroup analysis by clinical stage and Fuhrman nuclear grade (G) revealed that the deletion of *MPDZ* was associated with overall survival in stage III-IV (P=0.0337; Figure 2C), G 1-2 (P=0.0385; Figure 2D) and G 3-4 (P=0.0052; Figure 2E), but not in stage I-II (P=0.0903; Figure 2B). We also assessed the impact of *MPDZ* copy number on CSS using UCSC Xena software program. Whether in copy number or copy number with a gene level database, the CNV of *MPDZ* is significantly associated with worse CSS in patients with ccRCC (Supplementary Figure 2A–2C).

### The deletion of *MPDZ* is negatively correlated with its transcriptional expression from the TCGA cohort

**Figure 3:**
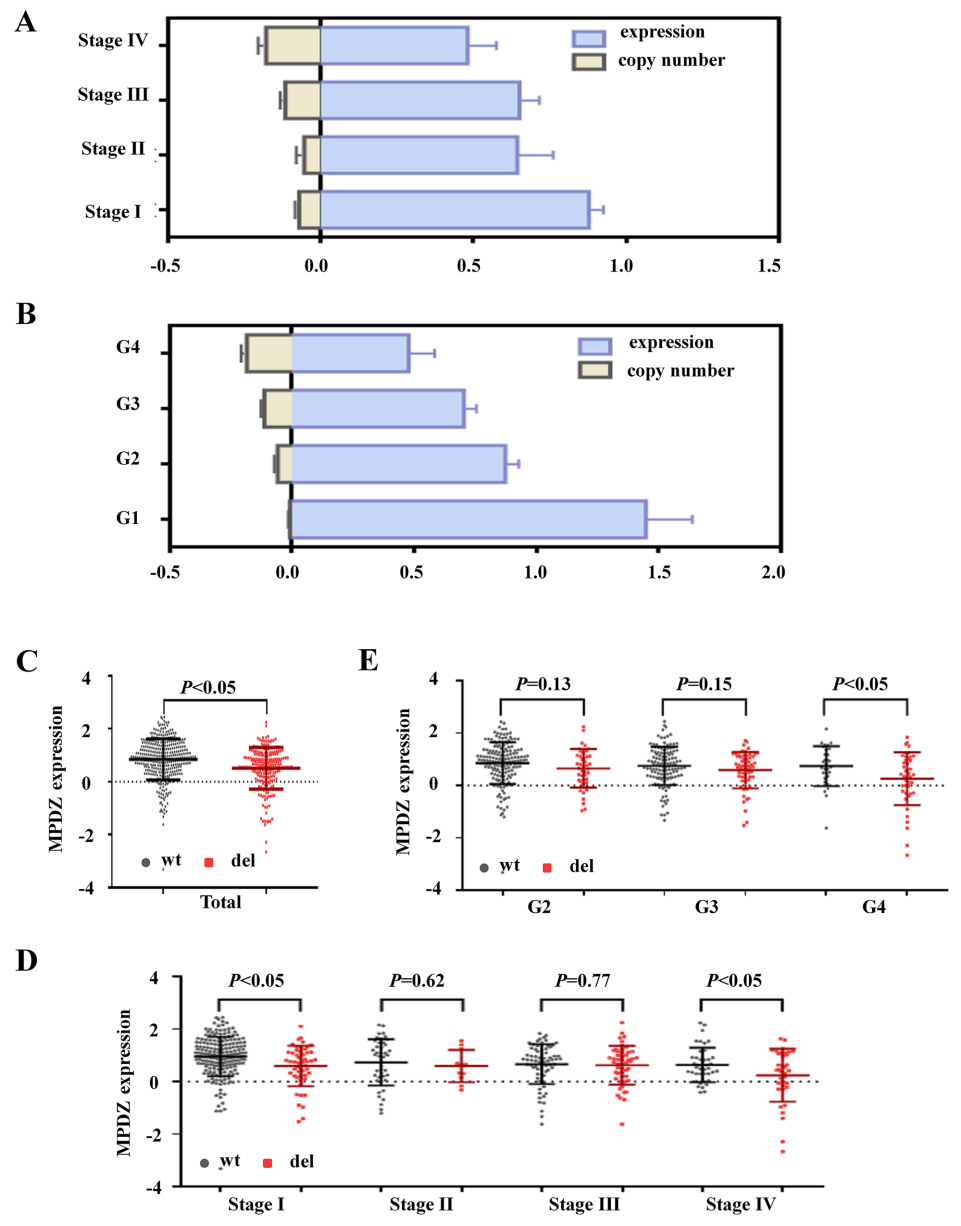
The MPDZ copy number was correlated with mRNA expression level in ccRCC from the TCGA Cohort The *MPDZ* mRNA expression and copy number were converted by index. The *MPDZ* mRNA expression and copy number in different clinical stages **(A)** and Fuhrman nuclear grades **(B)**. **(C)** The *MPDZ* mRNA expression in the wild-type group and deletion group. The *MPDZ* mRNA expression of wild-type group and deletion group show in different clinical stages **(D)** and Fuhrman nuclear grades **(E)**. wt, wild type; del, deletion; G, Fuhrman nuclear grades. Two-tailed Wilcoxon test.

To determine the association between the deletion and the expression of *MPDZ*, a correlation analysis was performed based on the 502 ccRCC tissue samples with both gene expression and deletion information. The *MPDZ* transcriptional expression in the low copy number group was significantly lower than that in high copy number group along with changes in the clinical stage and Fuhrman nuclear grade (G), respectively (Figure 3A–3B). We further investigated *MPDZ* expression levels between the deletion and wild-type group. Consistent with previous results, the expression of *MPDZ* was significantly downregulated in the deletion group compared with the wild-type group (P<0.05, Figure 3C). Furthermore, compared with the wild-type group, the expression of *MPDZ* in the deletion group was significantly downregulated in stage I, stage IV and G4 (P<0.05; Figure 3D–3E), but not in stage II, stage III, G2 and G3, respectively (Figure 3D–3E).

### *MPDZ* expression is significantly downregulated and associated with the poor survival of ccRCC patients from the TCGA cohort

**Figure 4:**
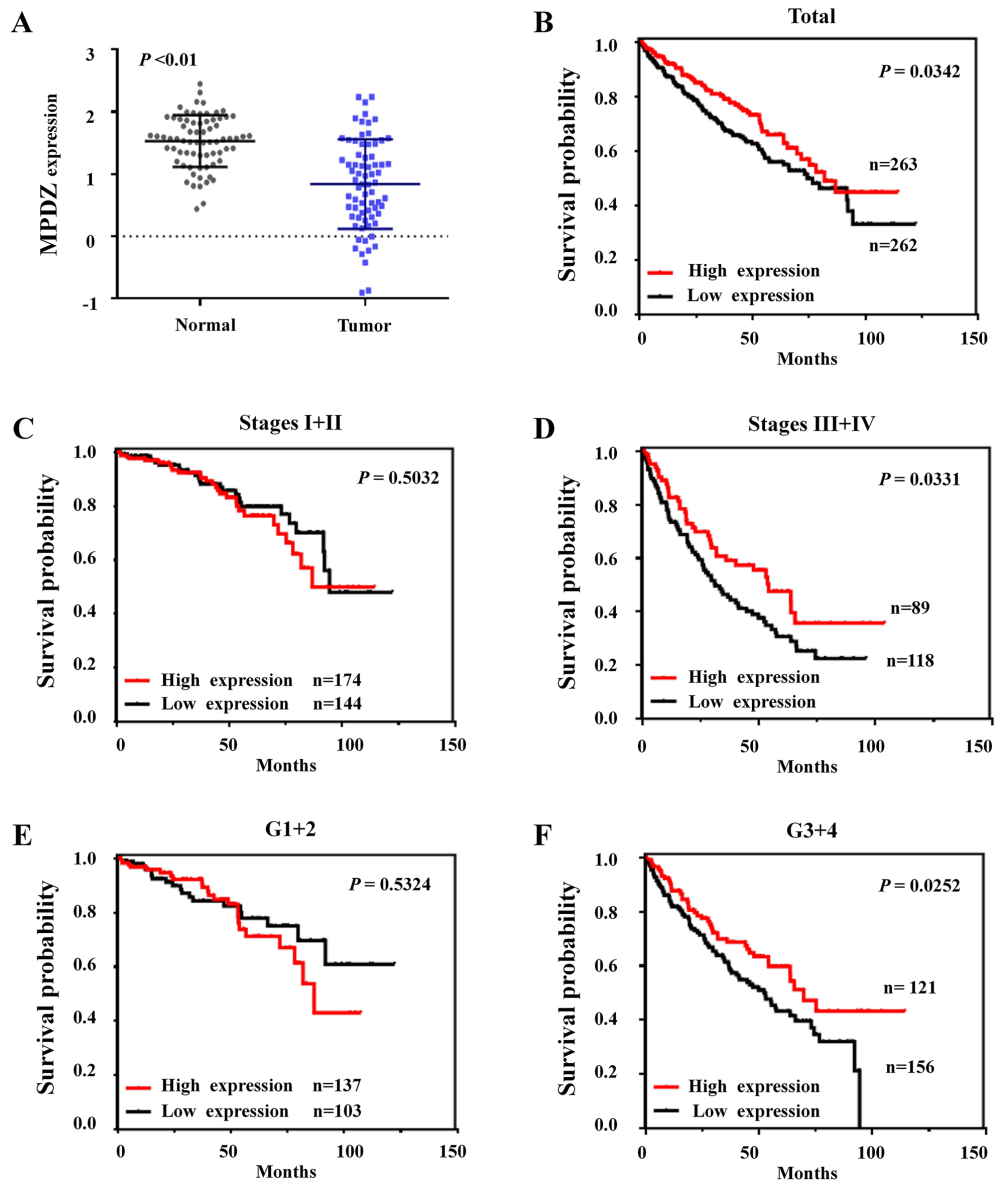
Expression patterns of MPDZ in ccRCC and its clinical significance in the TCGA Cohort **(A)** *MPDZ* expression is significantly downregulated in ccRCC tissues. Two-tailed Wilcoxon test. **(B)** Kaplan-Meier survival curves showed that patients with low *MPDZ* expression had poorer survival than those with high *MPDZ* expression. **(C)** Kaplan-Meier curves of *MPDZ* expression in patients with AJCC stage I and II. **(D)** Kaplan-Meier curves of *MPDZ* expression in patients with AJCC stage III and IV. **(E)** Kaplan-Meier curves of *MPDZ* expression in patients with Fuhrman nuclear grade 1 and 2. **(F)** Kaplan-Meier curves of *MPDZ* expression in patients with Fuhrman nuclear grade 3 and 4.

Because the deletion of *MPDZ* is an important regulation mechanism for its transcriptional expression, we further investigated *MPDZ* expression levels in 72 carcinoma tissues and adjacent tissues. *MPDZ* expression was significantly downregulated in ccRCC tissues compared with adjacent tissues (P<0.01, Figure 4A). Then, we compared the relationship between the expression of *MPDZ* and clinical characteristics in 525 patients with the clinical pathologic parameters available. Using median gene expression values as the cutoff, we found that low expression of *MPDZ* was significantly correlated with Fuhrman nuclear grade (P=0.0024), AJCC stage (P=0.0087), pathological T stage (P=0.0078), and pathological M stage (P=0.0357), but not with age, gender, total lymph nodes, lymph node status and pathological N stage (Table 2). A subgroup analysis by pathological T stage revealed that the low expression of *MPDZ* in T1 was significantly different from T2, T3 and T4 (P<0.05), but not in any other two groups.

**Table 2:** Clinical factor and MPDZ expression in ccRCC patients

Clinical factor	Numbers	MPDZ expression status		χ2	*P* value
		High	Low		
**Total**	525	263	262		
***Age (year)***				2.36	0.1243
< 60	240	129	111		
≥ 60	285	134	151		
***Gender***				1.14	0.2866
male	341	165	176		
female	184	98	86		
***Total lymph nodes***				0.05	0.8160
< 10	99	41	58		
≥ 10	32	14	18		
Unknown	394	208	186		
***Lymph node status***				2.80	0.0944
Negative	111	50	61		
Positive	17	4	13		
Unknown	397	209	188		
***Nuclear grade***				9.24	0.0024
1+2	240	137	103		
3+4	277	121	156		
other	8	5	3		
***AJCC stage***				6.89	0.0087
I +II	318	174	144		
III+IV	207	89	118		
***Pathological type***					
T (primary tumor)				11.90	0.0078
T1	267	152	115		
T2	68	27	41		
T3	179	81	98		
T4	11	3	8		
N (regional lymph nodes)				3.07	0.2159
N0	237	119	118		
N1	17	5	12		
NX	271	139	132		
M (distant metastases)				6.67	0.0357
M0	421	221	200		
M1	79	29	50		
MX	25	13	12		

Moreover, the clinical significance of *MPDZ* expression in ccRCC patients was assessed by performing a meta-analysis of the association of *MPDZ* gene expression with outcomes among ccRCC patients. We observed that the low expression of *MPDZ* was significantly associated with poor survival in patients with ccRCC (P=0.0342; Figure 4B). A subgroup analysis clinical stage and Fuhrman nuclear grade (G) revealed that *MPDZ* was associated with overall survival in stage III-IV (P=0.0331; Figure 4D) and G 3-4 (P=0.0252; Figure 4F), but not in stage I-II (P=0.5032; Figure 4C) and G 1-2 (P=0.5324; Figure 4E). To further confirm these results, we subsequently performed a meta-analysis using the UCSC Xena software program. Consistent with our results, low *MPDZ* expression levels are associated with poor overall survival in ccRCC patients (Supplementary Figure 3A–3C).

### Low MPDZ expression is associated with poor survival of ccRCC patients

**Figure 5:**
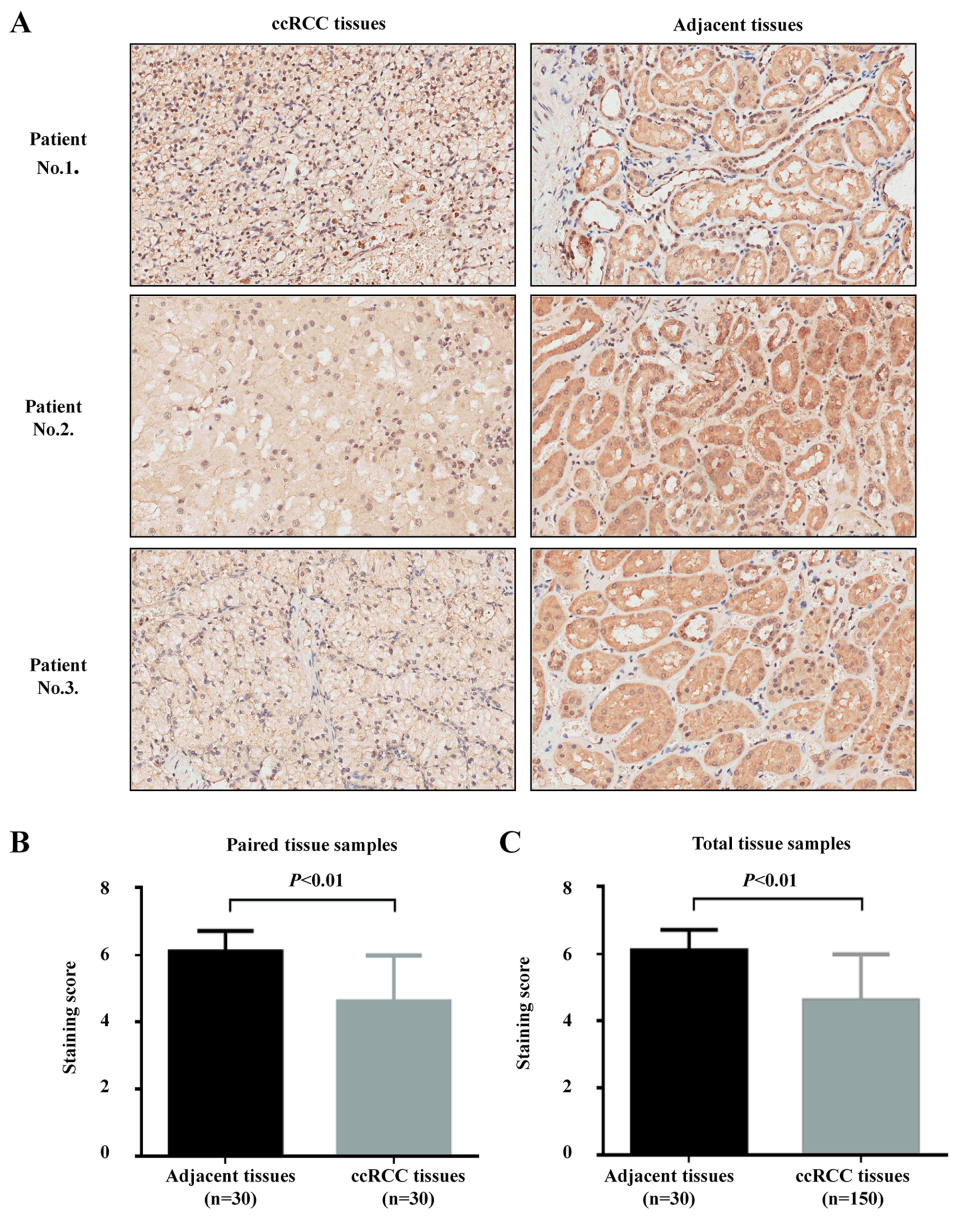
MPDZ expression is significantly downregulated in ccRCC tissues **(A)** Immunohistochemistry (IHC) analysis of *MPDZ* expression levels in adjacent and ccRCC tissues. *MPDZ* is highly expressed in adjacent tissues. Immunohistological staining assays were performed with an anti-*MPDZ* antibody (diaminobenzidine (DAB) staining, Magnification, ×200.). **(B)** *MPDZ* expression is significantly downregulated in ccRCC tissues according to the paired samples with staining score. **(C)** *MPDZ* expression is significantly downregulated in ccRCC tissues according to the total samples with staining score. Two-tailed Wilcoxon test.

**Figure 6:**
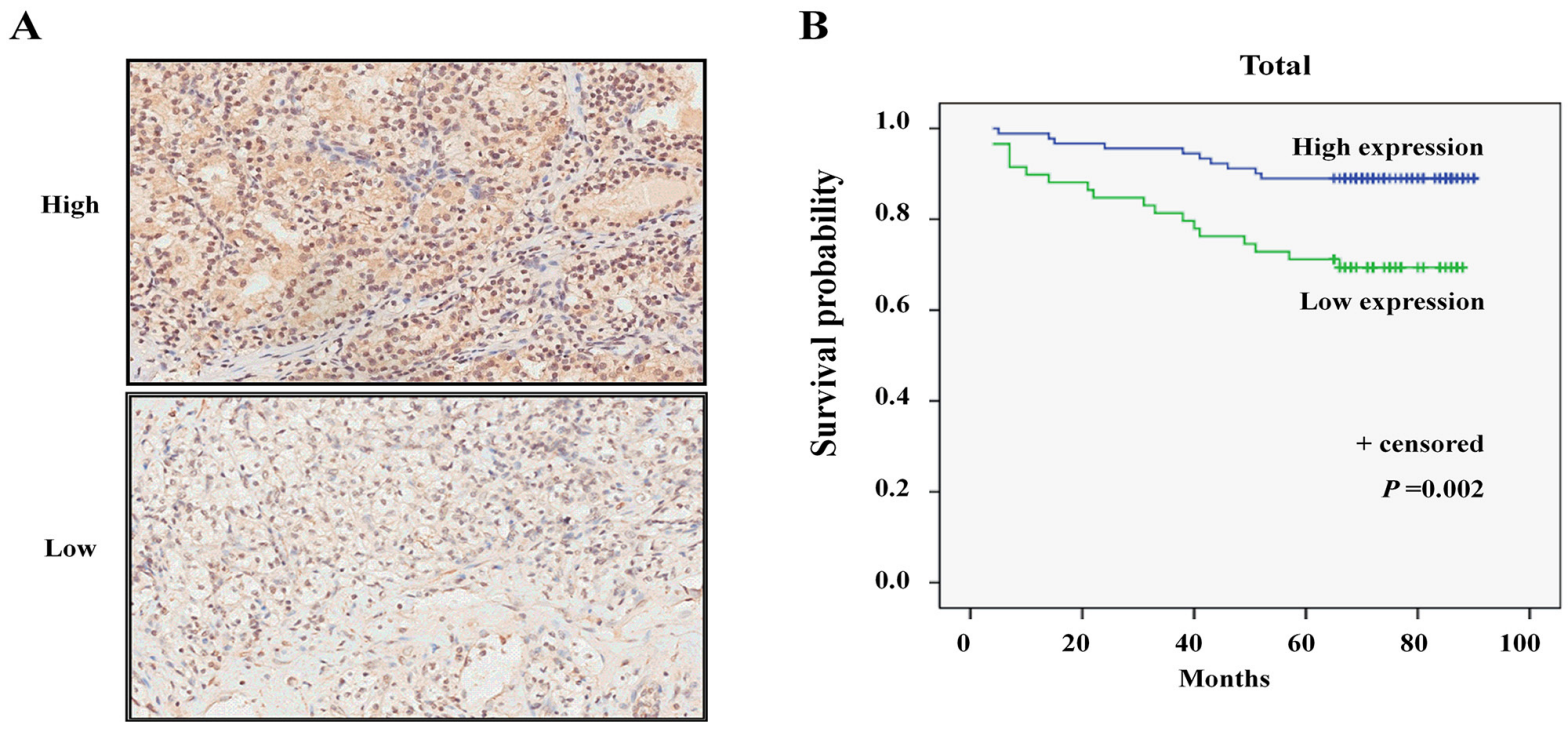
Low expression of MPDZ is significantly associated with poor survival in patients with ccRCC **(A)** The different *MPDZ* expression levels in ccRCC tissues by Immunohistochemistry (IHC) analysis. Immunohistological staining assays were performed with an anti-*MPDZ* antibody (diaminobenzidine (DAB) staining, Magnification, ×200.). **(B)** Kaplan–Meier survival curves were shown that patients with low MPDZ expression had poorer survival than those with high MPDZ expression.

To further evaluate the clinical significance of MPDZ, immunohistochemical analysis was performed in a tissue microarray of 150 ccRCC tissues and 30 adjacent tissues. We found that MPDZ was expressed at low levels in ccRCC tissues and expressed at high levels in adjacent tissue sample from different patients (Figure 5A). Compared with adjacent tissues, MPDZ expression was significantly downregulated in both paired (P<0.01; Figure 5B) and unpaired (P<0.01; Figure 5C) ccRCC tissues. In order to clarify whether MPDZ protein expression associated with prognosis of ccRCC patients, we used Kaplan–Meier survival curves to determine overall survival in ccRCC patients. Results showed that ccRCC patients with low MPDZ expression had significantly shorter survival than those with high MPDZ expression (P = 0.002, log-rank test; Figure 6).

## DISCUSSION

Accumulating evidence has demonstrated that predictive and prognostic markers have been proposed to distinguish between poor and favorable risk ccRCC patients [9, 11, 12]. However, these molecular markers stratified the survival curves and discriminated between stage distributions only when they are examined in the progression of ccRCC, but not in the early stage and precancerous lesions. To our knowledge, this is the first analysis on the association between the deletion of *MPDZ* and outcomes in patients with ccRCC. We found that *MPDZ* has been frequently deleted and downregulated in ccRCC tissues, which was associated with poor outcomes of ccRCC patients. Due to MPDZ is a protective factor in the renal osmoadaptive response [15, 20], the deletion of *MPDZ* may accelerate the progress of ccRCC under the osmotic pressure. The detection of *MPDZ* deletion may be a high-efficiency predictive method for ccRCC tumorigenesis.

Genetic changes by CNV play an important role in ccRCC and are probably good molecular biomarkers for diagnosis and prognosis of patients [21, 22]. Recently, CNV silencing of many novel genes that function as putative tumor suppressor genes has been reported to contribute to human cancer [23, 24]. In this study, we found that *MPDZ* is a typical CNV gene that commonly showed a CNV status in ccRCC. In addition, we found that there was an unusually simple mutation of *MPDZ* in ccRCC, which hasn’t influence on patients’ survival (Supplementary Figure 4A–4C). Because the percentage of deletion has the highest proportion in the CNV of *MPDZ*, much of the concern among CNV should be focused on the deletion in that the deletion of *MPDZ* is negatively correlated with its transcriptional expression. These genetic changes suggest that *MPDZ* is a potential tumor suppressor gene in ccRCC, which provides a novel tumor molecular marker for diagnosis, prognosis and therapy of patients with ccRCC.

In addition, we also observed that the deletion of *MPDZ* is significantly associated with poor outcomes in patients with ccRCC. However, there are few MPDZ amplifications among patients (only 15 persons from the database), which limits the statistical power because of the small sample. Accounting for the deletion is a common form in the CNV of *MPDZ*, and the deletion of *MPDZ* can decrease its expression. Thus, the del vs. wt model can be considered a conservative estimation of the impact of *MPDZ* on ccRCC survival. We found that the deletion of *MPDZ* is associated with poor outcomes in patients with ccRCC. Epigenetic silencing by DNA methylation plays an important role in many novel genes. We further analyzed the state and potential role of *MPDZ* methylation in ccRCC patients. Interestingly, we found that *MPDZ* methylation was also associated with poor outcomes in patients with ccRCC (Supplementary Figure 5). It is a potential reason for *MPDZ* CNV and expression data did not completely consistent, to some extent. These data are fairly straightforward in offering adequate evidence to confirm the fact that the methylation level of *MPDZ* is negatively correlated with its transcriptional expression. It is necessary to do further experimental research of the associations between the DNA methylation and the expression of *MPDZ*.

To date, the function of human *MPDZ* gene with several transcript variants (Supplementary Figure 1B) has not been thoroughly addressed in the literature. Most of studies associated with the function of the *MPDZ* gene were focused on the cytoskeleton, protein complex formation, signal transduction, cell polarity, cell osmotic pressure reactions and the causes of severe congenital hydrocephalus [14, 15, 25-27]. However, several reports showed that *MPDZ* participated in nasopharyngeal carcinoma and breast cancer tumorigenesis [17, 18]. However, it should be emphasized that these findings did not show that *MPDZ* is an independent factor for the prognosis of the tumor. Nevertheless, there is no study on *MPDZ* gene function in ccRCC. In the present study, we found that *MPDZ* was frequently deleted and downregulated in ccRCC tissues, which was associated with poor survival of ccRCC patients. Immunohistochemical analysis further certified that MPDZ was expressed at low levels in ccRCC tissues compared with the adjacent tissues. Kaplan–Meier survival curves showed that ccRCC patients with low MPDZ expression had significantly shorter survival than those with high MPDZ expression. These suggested that *MPDZ* is an independent factor for the prognosis of ccRCC and may play an important role in ccRCC. Interestingly, there had the positive nuclear staining of MPDZ in partly tissue samples particularly in tumor samples. Thus, further experimental research on the associations between MPDZ and ccRCC is needed. We would also study the specific mechanism of MPDZ that has different subcellular localization in cancer in future research.

In summary, our study showed that the genetic silencing of the *MPDZ* gene by deletions was associated with poor outcomes in patients with ccRCC. It provides knowledge regarding the deletion of *MPDZ* and expression variations and supports the potential role of prognostic significance, which has important clinical diagnostic and therapeutic implications in ccRCC.

## MATERIALS AND METHODS

### Phylogenetic analysis

MEGA 4 software, which includes neighbor-joining (NJ), Maximum Likelihood (ML) and Bayesian Markov Chain Monte Carlo (MCMC) approaches, was applied to generate an evolutionary tree to explain the phylogenetic relationships of the *MPDZ* transcript variant 1–X16.

### Mutation analysis

The mutation and its survival analysis were used cBioPortal for Cancer Genomics (http://www.cbioportal.org/), which including Multiregion Sequencing of Clear Cell Renal Cell Carcinoma (IRC, Nat Genet 2014), Clear Cell Renal Cell Carcinoma (U Tokyo, Nat Genet 2013), Kidney Renal Clear Cell Carcinoma (BGI, Nat Genet 2012), Kidney Renal Clear Cell Carcinoma (TCGA, Provisional) and Kidney Renal Clear Cell Carcinoma (TCGA, Nature 2013) studies.

### TCGA cohort

The datasets that included information on gene expression and CNV in ccRCC patients were downloaded from the TCGA (http://tcgadata.nci.nih.gov). The CNV information of ccRCC patients was acquired from the files entitled “TCGA_KIRC_gistic2thd-2015-02-24” and “TCGA_KIRC_gistic2-2015-02-24.” The gene expression data was obtained from the files entitled “TCGA_KIRC_exp_HiSeqV2_PANCAN-2015-02-24.” After we extracted gene expression information from the genomic data and integrated the clinical and pathologic information through scientific matches, there were 606 tissue samples (534 ccRCC tissues and 72 normal tissues) with *MPDZ* gene expression information and 525 ccRCC patients with expression, survival and clinical pathologic parameters available. Based on a similar scheme, there were 520 ccRCC patients with *MPDZ* gene CNV information and 505 ccRCC patients with the clinical pathologic parameters available were obtained for the deletion of *MPDZ* and survival analysis. Additionally, we assessed 502 ccRCC patients simultaneously including *MPDZ* expression and DNA copy number information for correlation analysis. All analyses, including gene expression, survival and CNV incidence, were based on the above database.

### CNVs analysis

After extracted CNVs data, the analysis of CNVs was followed the TCGA publication guidelines (http://cancergenome.nih.gov/publications). The level 3 CNV of each sample was processed and normalized. The mean copy number estimates of segments overlapping the whole genome were obtained and used for the analysis. Genomic identification of significant targets in cancer (GISTIC) algorithm mean cut-offs were used to categorize the gene. The copy number data of each sample were discretized by binning the copy number calls as amplification or deletion using a threshold of >=1 for amplification and <=-1 for deletion. The formula (number of samples with amplification or deletion in a group)/(total number of samples in a group) were used to calculate the frequency of amplification or deletion.

### Tissue microarray (TMA) and immunohistochemistry

A TMA including 150 ccRCC tissues and 30 adjacent tissues with clinical and prognostic information was obtained from Shanghai Biochip Company Ltd of China. An antibody against MPDZ (SC-135504, Santa Cruz Biotechnology, Santa Cruz, CA, USA) was used to perform immunohistochemical staining in TMA chips as previously described [28]. Two pathologists independently reviewed all core biopsies. The immunostaining was considered positive when ≥10% of the tumour cells was immunoreactive. The intensity of staining was graded semi-quantitatively as negative (scored as 0), weak (1), moderate (2) or strong (3) positivity. The percentage of positive cells, as the extent of immunostaining, was quantified into five groups under microscope: <10% positive cells for 0; 10–25% positive cells for 1; 26–50% positive cells for 2; 51–75% positive cells for 3 and ≥ 76% positive cells for 4. Multiplying the percentage of positive staining and the intensity was used to define expression levels. A final staining score more than six was considered to be high expression.

### Kaplan-Meier plotter analysis

The prognostic value of the *MPDZ* gene in ccRCC patients was analyzed using the Kaplan–Meier method. The boundaries of high and low expression of *MPDZ* used the median gene expression value. The competing risk method was applied for CSS analysis. The TCGA datasets to adjust for clinical covariates (AJCC stage I and II vs. III and IV and grade 1 and 2 vs. 3 and 4) used multivariate competing risk models. The classification standards of the deceased patients were according to the previous study [29]. The UCSC Xena (https://genome-cancer.soe.ucsc.edu) was also used to analyze the associations between gene expression, methylation, CNV and survival in ccRCC patients.

### Statistical analysis

The relative standard errors of the mean normalized values were used for gene expression. The gene expression results between the two groups were evaluated using a two-tailed Wilcoxon test and Bonferroni corrected for multiple hypothesis testing. Spearman’s rank correlation was applied to analyze the correlation between gene expression and copy numbers. The difference in categorical variables was analyzed by Chi-square test. Multiple comparisons that a 2 × 2 contingency table of expected and observed deletion frequencies were used to analyze more than two groups, with a P-value cut-off set at 0.05 with stringent false discovery rate control (Bonferroni’s method). A two-sided P-value<0.05 was considered statistically significant. Statistical analyses were performed with the SPSS 19.0 software (SPSS, Inc., Chicago, IL, USA).

## SUPPLEMENTARY MATERIALS FIGURES



## Abbreviations

*MPDZ*: *multiple PDZ domain protein*
ccRCC: clear cell renal cell carcinoma
CNV: copy number variation
TCGA: the cancer genome atlas
