# Supplementary material for: Copy number variations and expression of MPDZ are prognostic biomarkers for clear cell renal cell carcinoma
Source: Oncotarget. 2017 Aug 12;8(45):78713–25. doi: 10.18632/oncotarget.20220 (PMC5667992; doi:10.18632/oncotarget.20220)
Supplement: Supplementary file 1 [file oncotarget-08-78713-s001.pdf]

Copy number variations and expression of MPDZ are prognostic biomarkers for clear cell renal cell carcinoma

SUPPLEMENTARY MATERIALS

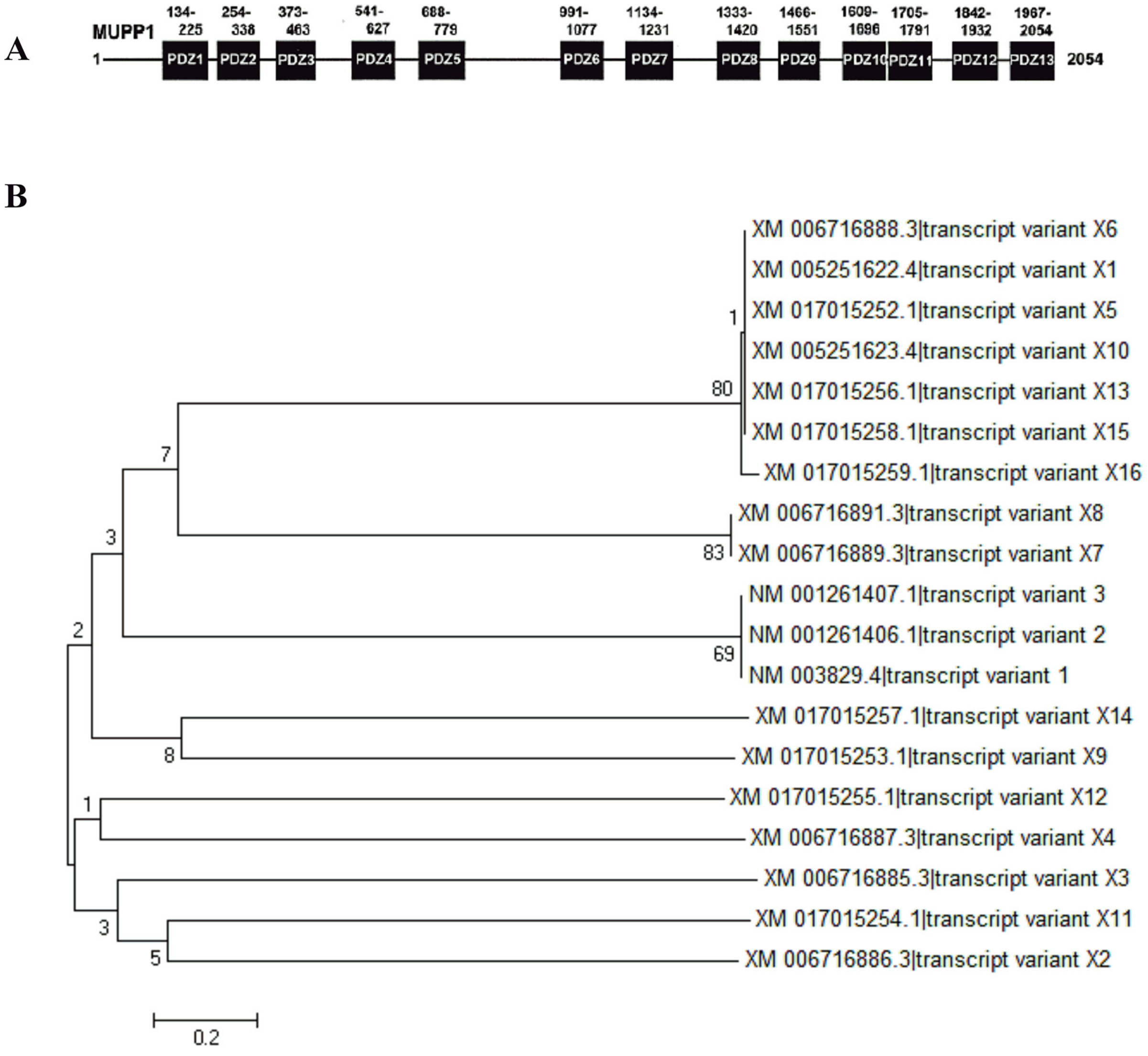

Supplementary Figure 1: The MPDZ protein structure and transcript variants. (A) The *MPDZ* gene encodes a protein of 2070 amino acids that contains thirteen PDZ domains. **(B)** The *MPDZ* gene has 19 transcript variants.

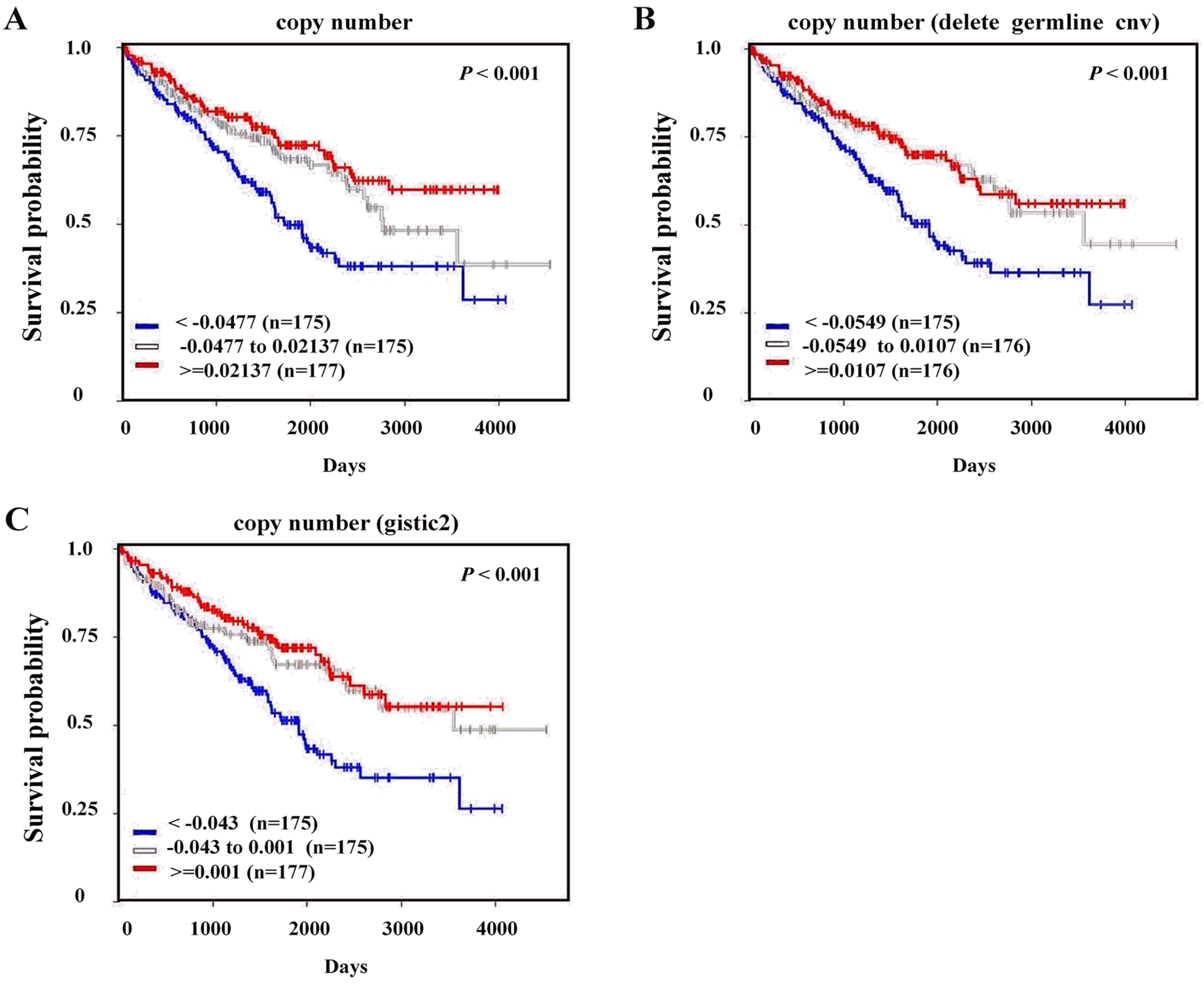

Supplementary Figure 2: The low DNA copy number of MPDZ was associated with poor outcomes in ccRCC. **(A)** Kaplan–Meier survival analysis based on the *MPDZ* copy number. **(B)** Kaplan–Meier survival analysis based on the *MPDZ* copy number (delete germline cnv). **(C)** Kaplan–Meier survival analysis based on the *MPDZ* copy number (gistic2).

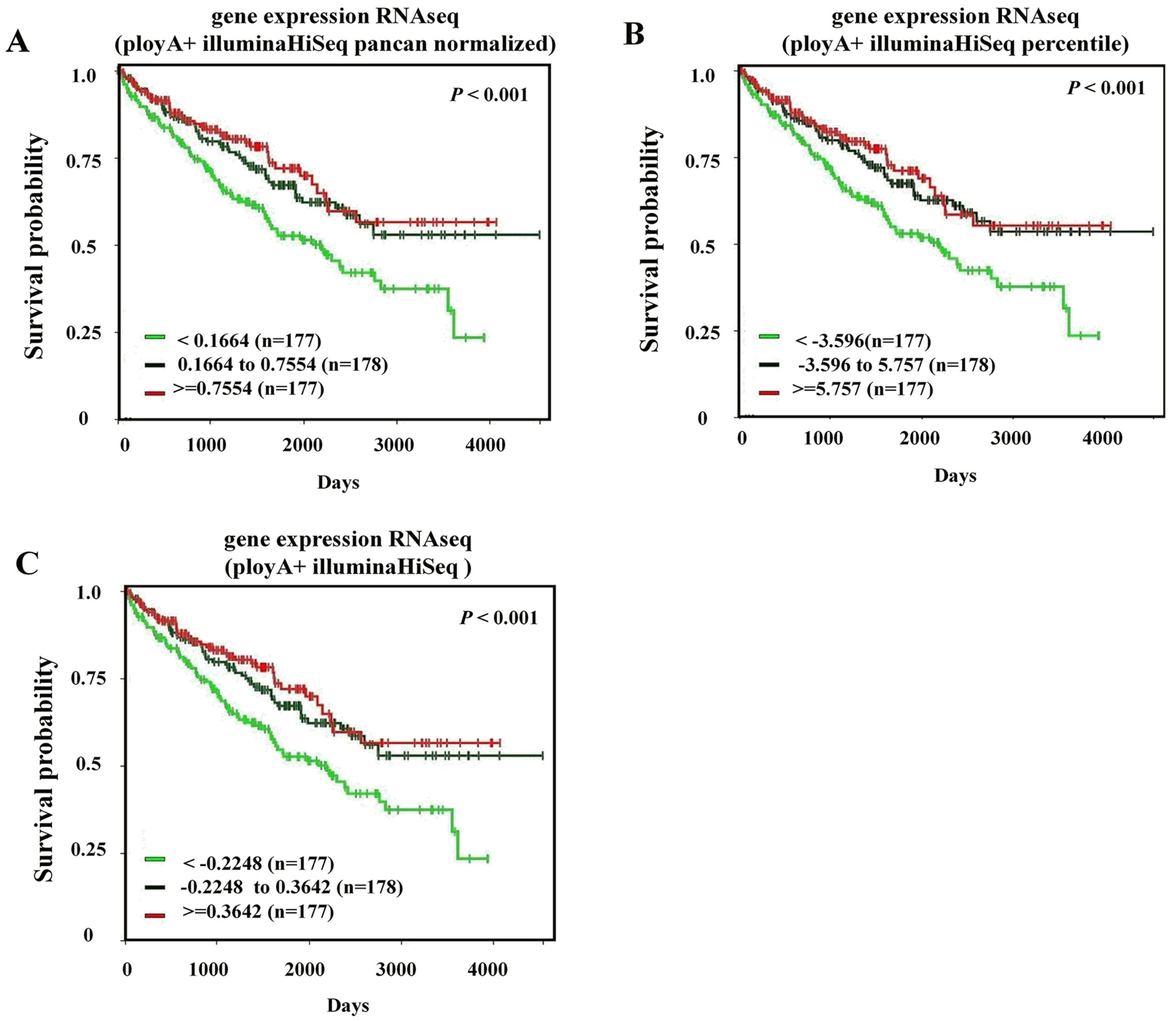

Supplementary Figure 3: MPDZ downexpression was associated with poor survival in ccRCC. **(A)** Kaplan–Meier survival analysis based on *MPDZ* gene expression via RNAseq (ployA+ illuminaHiSeq pancan normalized). **(B)** Kaplan–Meier survival analysis based on *MPDZ* gene expression via RNAseq (ployA+ illuminaHiSeq percentile). **(C)** Kaplan–Meier survival analysis based on *MPDZ* gene expression via RNAseq (ployA+ illuminaHiSeq).

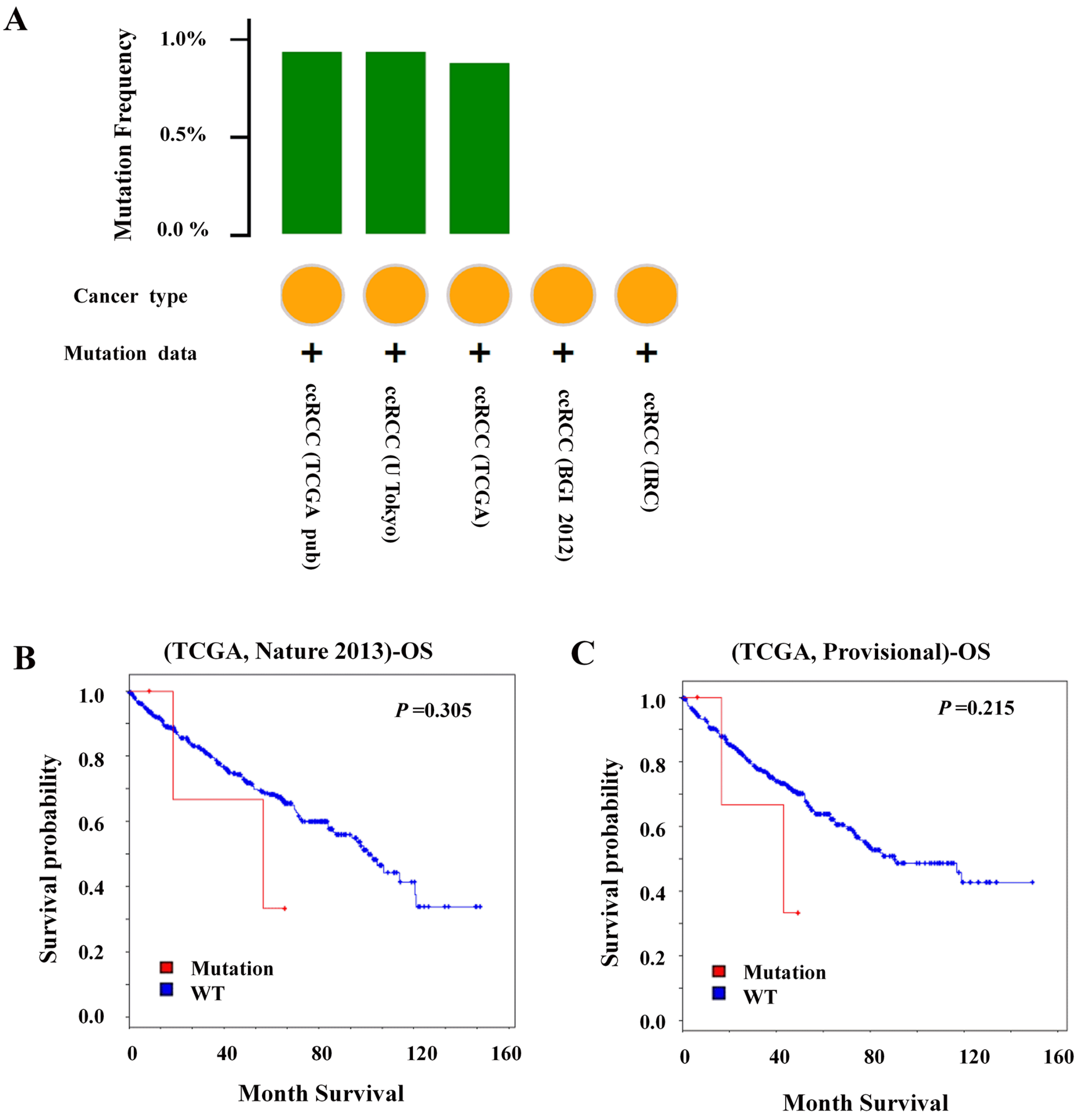

Supplementary Figure 4: The effect of MPDZ mutation on ccRCC patients' survival. (A) The incidence of *MPDZ* mutation in different ccRCC studies. **(B)** Kaplan–Meier curves of mutations in patients by "Kidney Renal Clear Cell Carcinoma (TCGA, Nature 2013)" study. **(C)** Kaplan–Meier curves of mutations in patients by "Kidney Renal Clear Cell Carcinoma (TCGA, Provisional)" study.

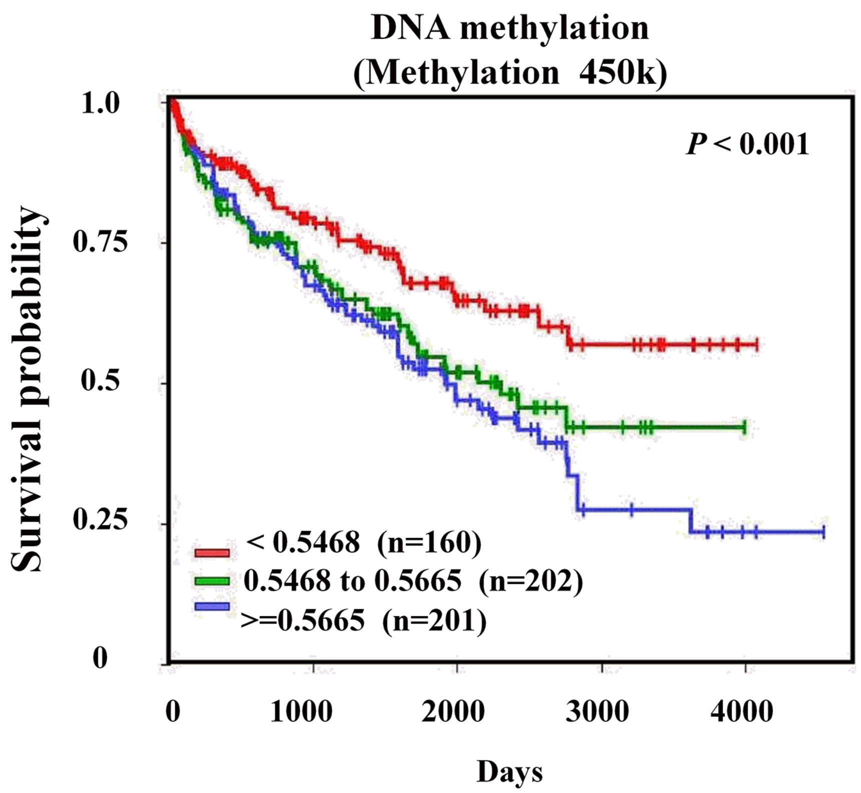

Supplementary Figure 5: The effect of MPDZ methylation on ccRCC patients' survival. Kaplan–Meier survival analysis based on the *MPDZ* methylation (450 k).
